# Role and mechanism of miR-222 in arsenic-transformed cells for inducing tumor growth

**DOI:** 10.18632/oncotarget.7525

**Published:** 2016-02-20

**Authors:** Min Wang, Xin Ge, Jitai Zheng, Dongmei Li, Xue Liu, Lin Wang, Chengfei Jiang, Zhumei Shi, Lianju Qin, Jiayin Liu, Hushan Yang, Ling-Zhi Liu, Jun He, Linlin Zhen, Bing-Hua Jiang

**Affiliations:** ^1^ State Key Laboratory of Reproductive Medicine, Department of Pathology, and Collaborative Innovation Center for Cancer Personalized Medicine, Cancer Center, Nanjing Medical University, Nanjing, Jiangsu, China; ^2^ Department of Breast and Thyroid Surgery, Huai'an First People's Hospital, Huai'an, Jiangsu, China; ^3^ Center of Clinical Reproductive Medicine, Jiangsu Province Hospital, Nanjing, Jiangsu, China; ^4^ Ninggao Personalized Medicine and Technology Innovation Center, Nanjing, Jiangsu, China; ^5^ Division of Population Science, Department of Medical Oncology, Thomas Jefferson University, Philadelphia, PA, USA; ^6^ Department of Pathology, Anatomy and Cell Biology, Thomas Jefferson University, Philadelphia, PA, USA

**Keywords:** BEAS-2B cells, miR-222, PTEN, ARID1A, tumor growth

## Abstract

High levels of arsenic in drinking water, soil, and air are associated with the higher incidences of several kinds of cancers worldwide, but the mechanism is yet to be fully discovered. Recently, a number of evidences show that dysregulation of microRNAs (miRNAs) induces carcinogenesis. In this study, we found miR-222 was upregulated in arsenic-transformed human lung epithelial BEAS-2B cells (As-T cells). Anti-miR-222 inhibitor treatment decreased cell proliferation, migration, tube formation, and induced apoptosis. In addition, anti-miR-222 inhibitor expression decreased tumor growth *in vivo*. We also found that inhibition of miR-222 induced the expression of its direct targets ARID1A and phosphatase and tensin homolog deleted on chromosome 10 (PTEN), and activated apoptosis of As-T cells in part through ARID1A downregulation. These results indicate that miR-222 plays an important role in arsenic-induced tumor growth.

## INTRODUCTION

Long-term exposure to arsenic can result in skin, liver, lung, and other types of cancer [1–3]. Arsenic was identified as an environmental carcinogen by the International Center for Research on Cancer in 1987 and the USA Environmental Protection Agency in the 1990s [4]. But new molecular mechanisms of arsenic-induced carcinogenesis remain to be elucidated.

A number of studies have shown a close relationship between microRNA (miRNA) and the occurrence of cancers [5, 6]. MiRNAs may play roles as tumor suppressors or oncogenes through their targets. The abnormal expression of miRNAs exists widely in tumor tissues. MiR-199a and miR-145 are commonly regarded as tumor suppressor genes in many cancers [7, 8]. It is reported that miR-222 is an oncogene that is upregulated in many cancers including hepatocellular carcinoma, cervical cancer, and gastric carcinoma. miR-222 can increase migration and proliferation of tumor cell, and inhibit apoptosis by regulating different targets such as PTEN [9], p27 [10], and TIMP3 [11]. Overexpression of miR-222 is correlated with the poor prognosis of non-small cell lung cancer (MSCLC) and miR-122 may be used as a biomarker for selecting the Tube formation assay patients who require especial attention [12].

Recently, arsenic has been found to regulate expression of some miRNAs. It was reported that several miRNAs were upregulated; while miR-199a, miR-200b, miR-164, and miR-171 were downregulated in response to arsenic treatment [13, 14]. It was also reported that miR-21 is upregulated by arsenic to induce angiogenesis and to enhance the invasive potential of transformed cells [15, 16]. Our previous study showed that miR-199a is downregulated in arsenic-transformed (As-T) cells, and arsenic inhibited miR-199a expression for decreasing angiogenesis [17]. But the roles and mechanisms of miRNAs in arsenic-induced tumor growth are not fully elucidated.

The present study addresses the following questions: (1) the expression and the role of miR-222 in cell proliferation, migration and tube formation in cells; (2) the role of miR-222 in tumor growth; and (3) the functional relevant target(s) of miR-222 in As-T cells.

## RESULTS

### miR-222 expression is upregulated in As-T cells

**Figure 1:**
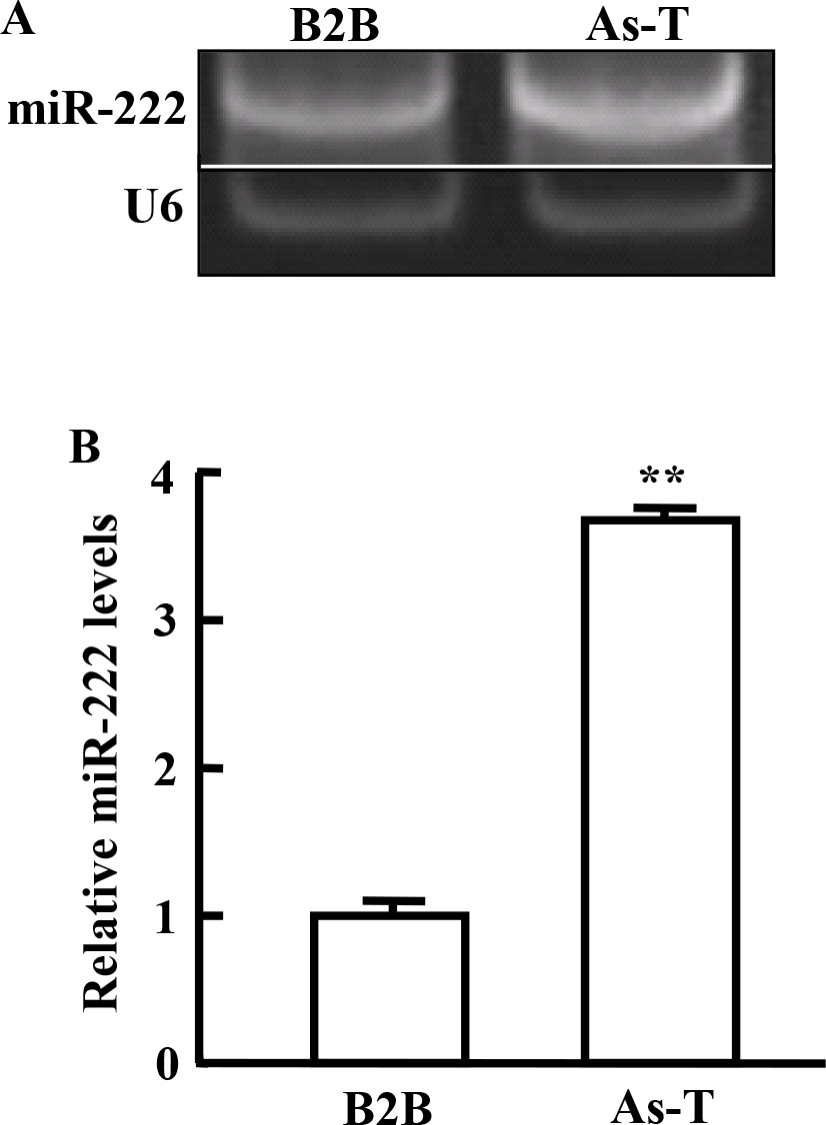
miR-222 expression is upregulated in As-T cells The expression levels of miR-222 were determined using RT-PCR (**A**) and RT-qPCR (**B**) in As-T cells and BEAS-2B (B2B) cells. The expression levels of miR-222 were normalized to U6 snRNA levels. Data are presented as mean ± SE. **indicates significant difference compared to that of control cells (*P* < 0.01).

Abnormal expression of miRNAs in tumors is an important feature of tumor development. Our result showed that miR-222 expression was upregulated the most in As-T cells using miRNA microarray analysis (data not shown). Similar results were confirmed using RT-PCR and RT-qPCR analysis, showing 4-fold higher levels of miR-222 in As-T cells than in B2B cells (Figure 1A and 1B).

### Treatment of cells using anti-miR-222 inhibitor decreases cell proliferation, migration, tube formation, and tumor growth

**Figure 2:**
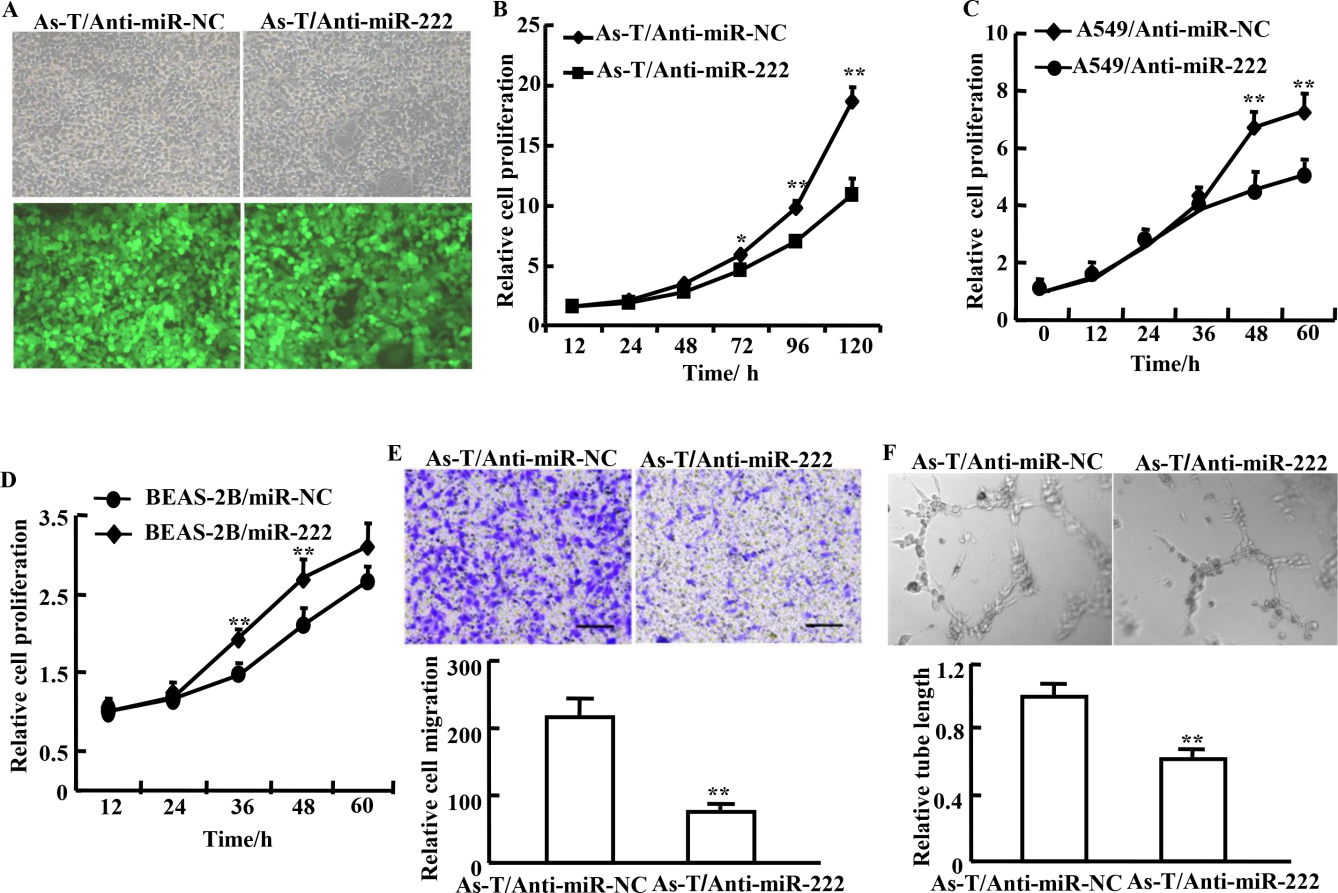
Treatment of cells using anti-miR-222 inhibitor decreases cell proliferation, migration, and tube formation (**A**) As-T cells were infected with lentivirus carrying anti-miR-222 inhibitor and GFP or a negative control and GFP according to the manufacturer's instruction. The cells with green fluorescence represent stably overexpressing miR-222 inhibitor (As-T/Anti-miR-222) or negative control As-T/Anti-miR-NC. (**B**) The CCK-8 assay was performed according to the manufacturer's instruction to analyze proliferation of As-T cells expressing anti-miR-222 inhibitor (As-T/Anti-miR-222) or negative control (As-T/Anti-miR-NC). Proliferation rates were determined at 12, 24, 48, 72, 96, and 120 h after the seeding. (**C, D**) Similar experiments as above were performed in A549 cells (C), and in BEAS-2B cells (D) at 12, 24, 48 and 60 h. (**E**) Migration of As-T cells overexpressing anti-miR-222 inhibitor (As-T/anti-miR-222) or negative control (As-T/miR-NC) using Transwell assay. (**F**) As-T cells at 90% confluence stably overexpressing anti-miR-NC or anti-miR-222 inhibitor were cultured in 4 mL serum–reduced medium (1% FBS) in a 10 cm dish for 24 h, then the conditioned medium was collected for tube formation assay using HUVECs as we previously described. Data are represented as mean ± SE from five replicates from each treatment. * and ** indicate significant difference compared to the control (*P* < 0.05 and *P* < 0.01, respectively). Scale bar: 500 μm. Magnification: ×400. Scale bar: 50 μm.

**Figure 3:**
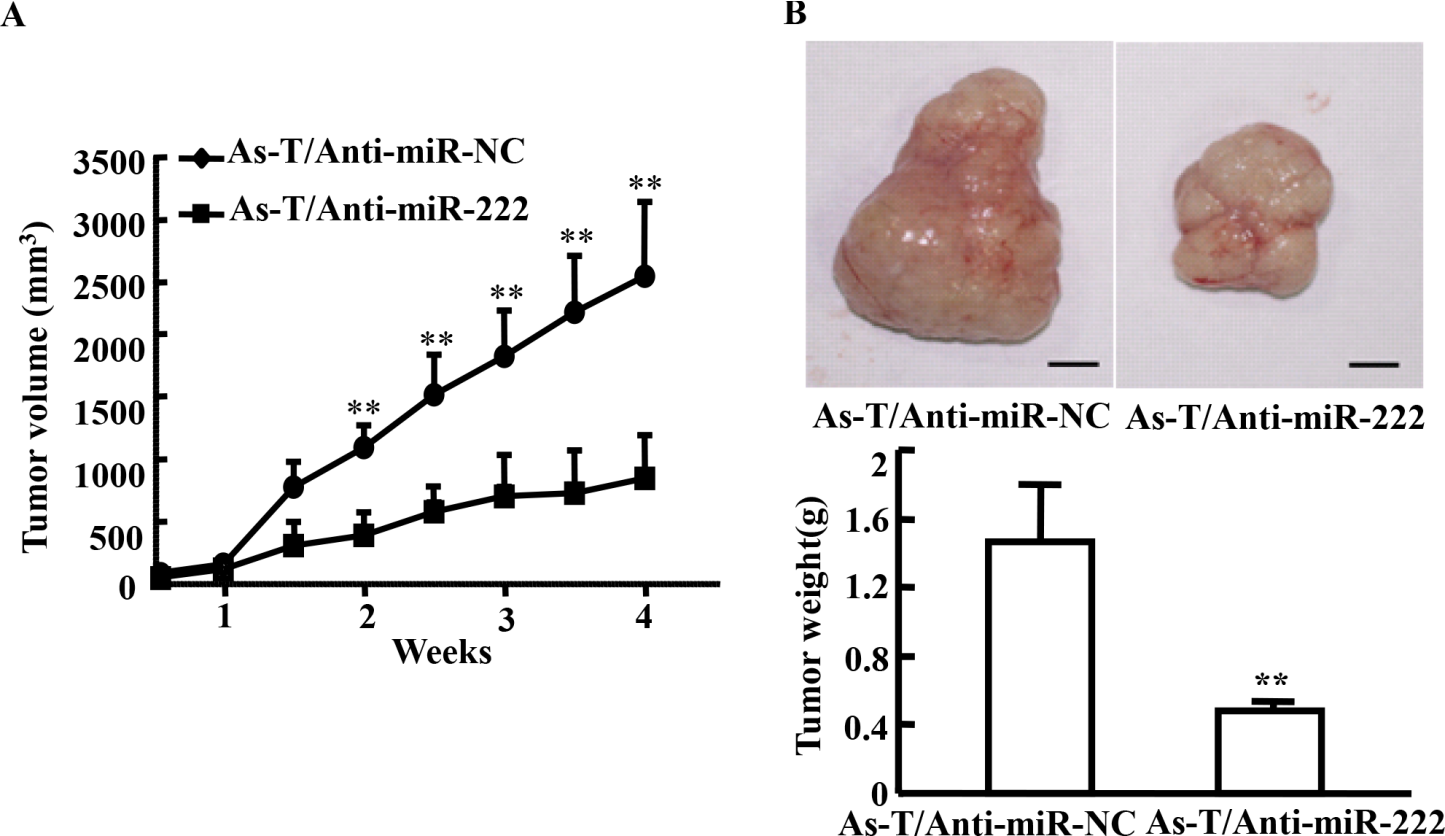
Expression of anti-miR-222 inhibitor in cells decreases As-T cells-induced tumor growth *in vivo* (**A**) BALB/c nude mice (5–6 week old) were subcutaneously injected with 5 × 10^6^ As-T cells expressing anti-miR-NC or anti-miR-222 inhibitor. Sizes of tumors were measured two times every week after they were visible. Volumes of tumors were calculated using the formula 0.5 × length × width^2^ [18]. (**B**) The tumors were resected and weighed after 28 days of the cell injection. Data were presented as mean ± SD. **indicates significant difference at *P* < 0.01.

To study the potential biological function of miR-222 in As-T cells, the cells stably overexpressing miR-222 inhibitor (As-T/Anti-miR-222) or negative control (As-T/Anti-miR-NC) were established, confirming the successful establishment of the stable cells (Figure 2A). The proliferation rates of As-T cells with forced expression of anti-miR-222 inhibitor were significantly decreased after 72 h post cell seeding compared with the control cells (Figure 2B). Similarly, anti-miR-222 inhibitor treatment significantly decreased cell proliferation of NSCLC A549 cells, whereas miR-222 precursor significantly increased cell proliferation of BEAS-2B, the immortal normal bronchial epithelial cells (Figure 2C and 2D). Cell migration was analyzed using Transwell assay. The result showed that cell migration was decreased by more than 2-fold in As-T cells transfected with anti-miR-222 inhibitor (Figure 2E). The tube formation was also significantly decreased by anti-miR-222 inhibitor treatment (Figure 2F). Finally, to further investigate the role of miR-222 in tumor growth *in vivo*, ectopic transplantation model of As-T cells in nude mice was employed. Stable cell lines, As-T/Anti-miR-NC and As-T/Anti-miR-222 cells were obtained and subcutaneously injected into posterior blanks of BALB/c nude mice, respectively. Each group includes ten mice. Tumor volumes were monitored twice per week when the tumors are visible. Compared to the control group, the tumor volume of anti-miR-222 inhibitor group was significantly smaller by Week 2 (*P* < 0.05, Figure 3A). Nude mice were sacrificed four weeks after implantation, and xenografts were trimmed out. The tumor sizes of anti-miR-222 inhibitor group were much smaller than that of control group (Figure 3B, top). Consistent with tumor size, the tumor weight of anti-miR-222 inhibitor group was decreased to 30% of control group (Figure 6B, tmiR-222 levels in As-T cells is sufficient to attenuate tumor growth *in vivo*.

### miR-222 directly targets PTEN for inhibiting its expression

**Figure 4:**
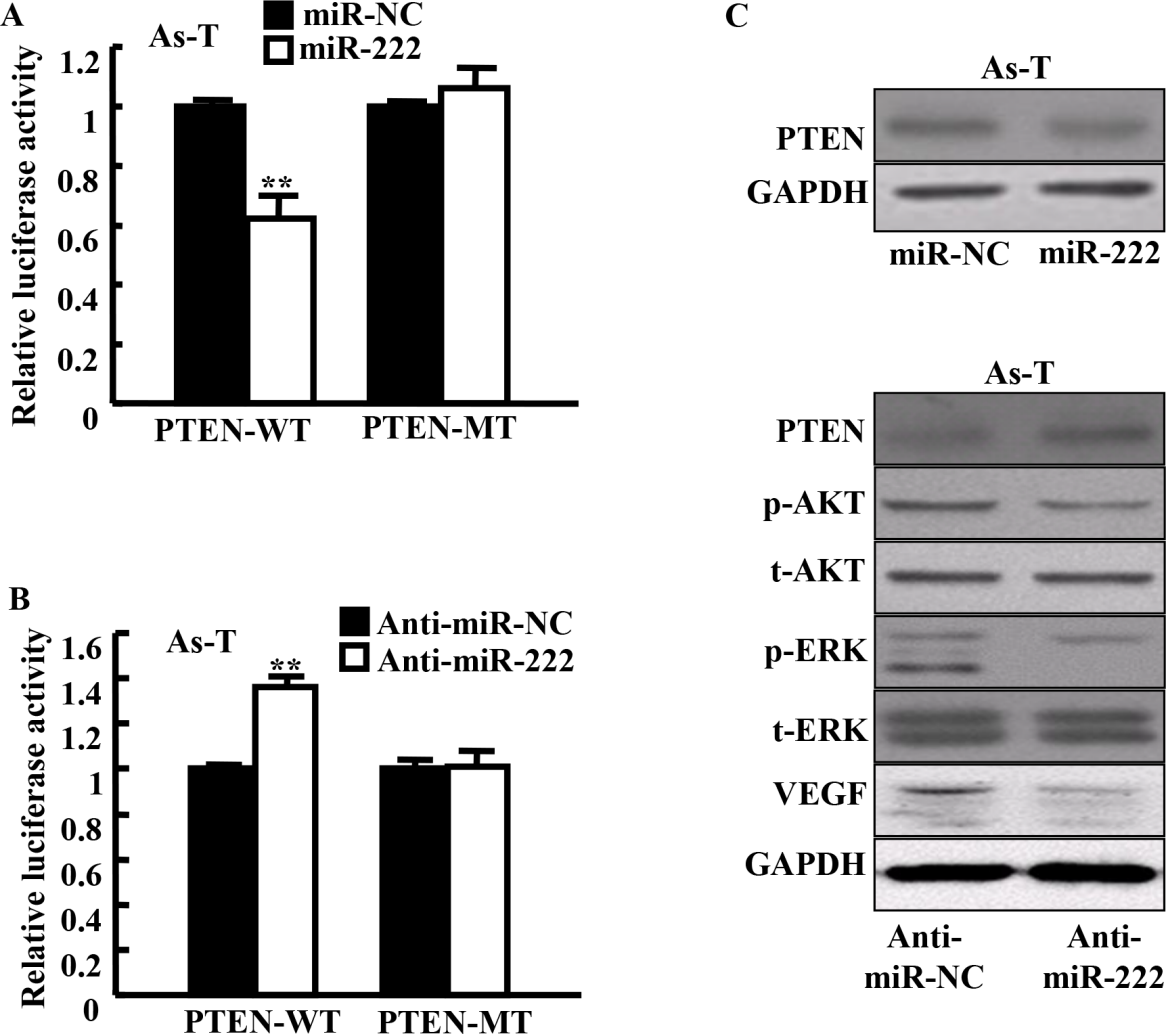
miR-222 directly targets PTEN for activating several downstream signal molecules (**A** and **B**) PTEN wild-type and mutant 3′-UTR region reporter activities were assayed as in the Methods. Data are presented as mean ± SE. **indicates significant difference compared to those of control cells (*P* < 0.01). (**C**) The levels of PTEN protein and its several downstream signal proteins in these cells were determined using Western blotting at 48 h after the transfection. Representative blotting images are shown.

It has been reported that PTEN is a target of miR-122 [9]. To verify whether miR-222 directly targets PTEN, PTEN 3′-UTR sequences containing putative binding sites of wild type (WT) or the mutant one (mut) were cloned into pMIR-REPORTER vector. As-T cells were cotransfected with reporter plasmid (PTEN-WT or PTEN-mut) and miR-222 precursor or negative control (miR-NC). Luciferase assay showed that the luciferase activities of wild type PTEN 3′-UTR reporter were inhibited by 35% in As-T cells over-expressing miR-222. On the opposite, inhibition of miR-222 by its inhibitor increased the luciferase activities of wild type reporter by nearly 50% in As-T cells (Figure 4A and 4B). Neither miR-222 nor miR-222 inhibitor affected the luciferase activities of mutant reporters. This result suggests that miR-222 inhibits PTEN expression through the seed sequence at its 3′-UTR region. Further study by immunoblotting assay showed that forced expression of miR-222 greatly inhibited the expression levels of PTEN, while blockade of endogenous expression of miR-222 upregulated PTEN levels for decreasing downstream signaling molecule activation of PTEN: p-AKT, p-ERK, and VEGF levels (Figure 4C).

### miR-222 directly targets ARID1A for inhibiting its expression

**Figure 5:**
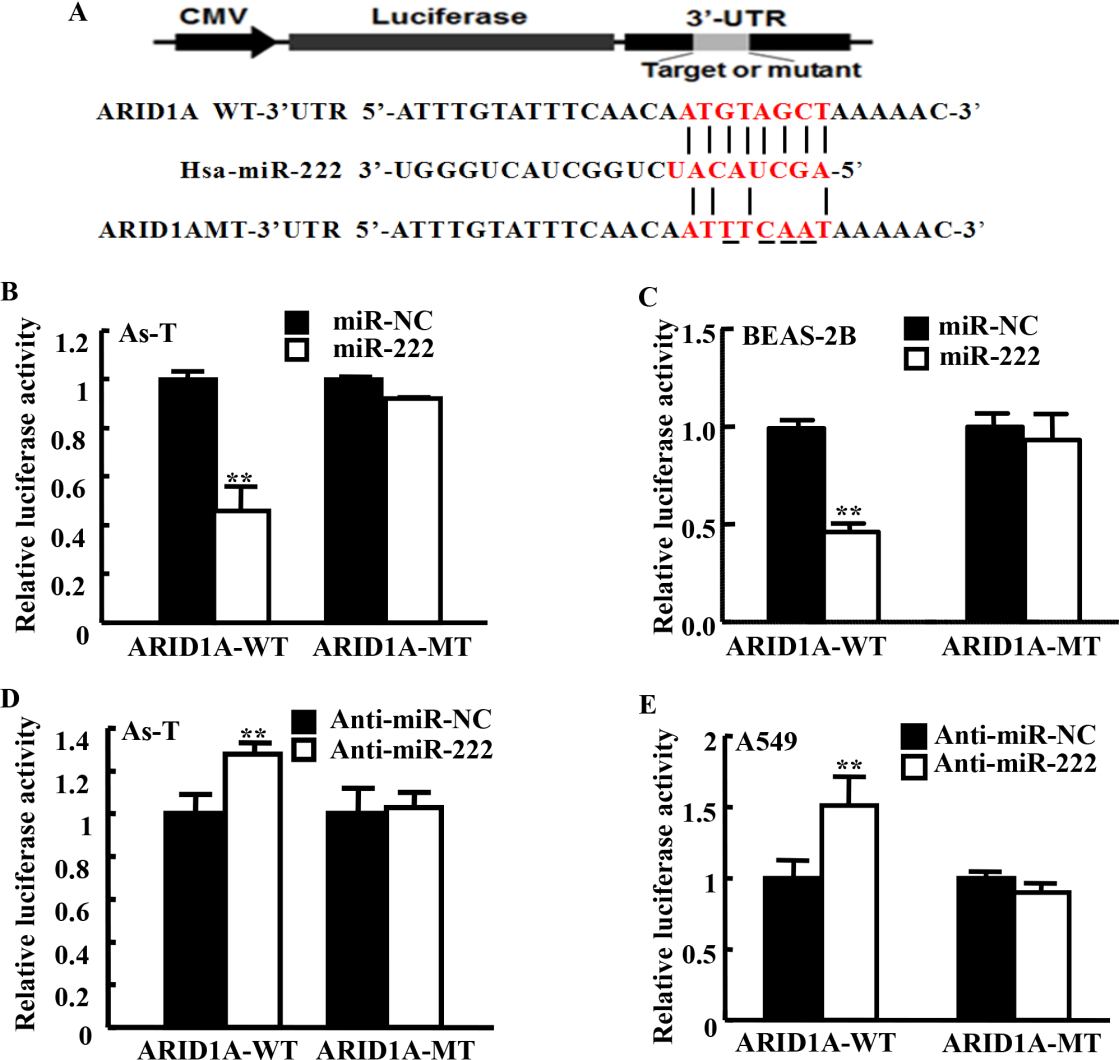
MiR-222 directly targets ARID1A through the seed sequence at its 3′-UTR region (**A**) Putative seed-matching sites or mutant sites of ARID1A 3′-UTR region with miR-222. (**B, C**) These ARID1A 3′-UTR luciferase reporter constructs were cotransfected using miR-222 mimics in As-T cells (B) or in BEAS-2B cells (C), and analyzed as above. (**D, E**) These reporter constructs were cotransfected with anti-miR-222 inhibitor in As-T cells (D) or in A549 cells (E), and analyzed as above. Data are presented as mean ± SE from three independent experiments with three replicates per experiment. **indicates significant difference compared to that of control cells (*P* < 0.01).

**Figure 6:**
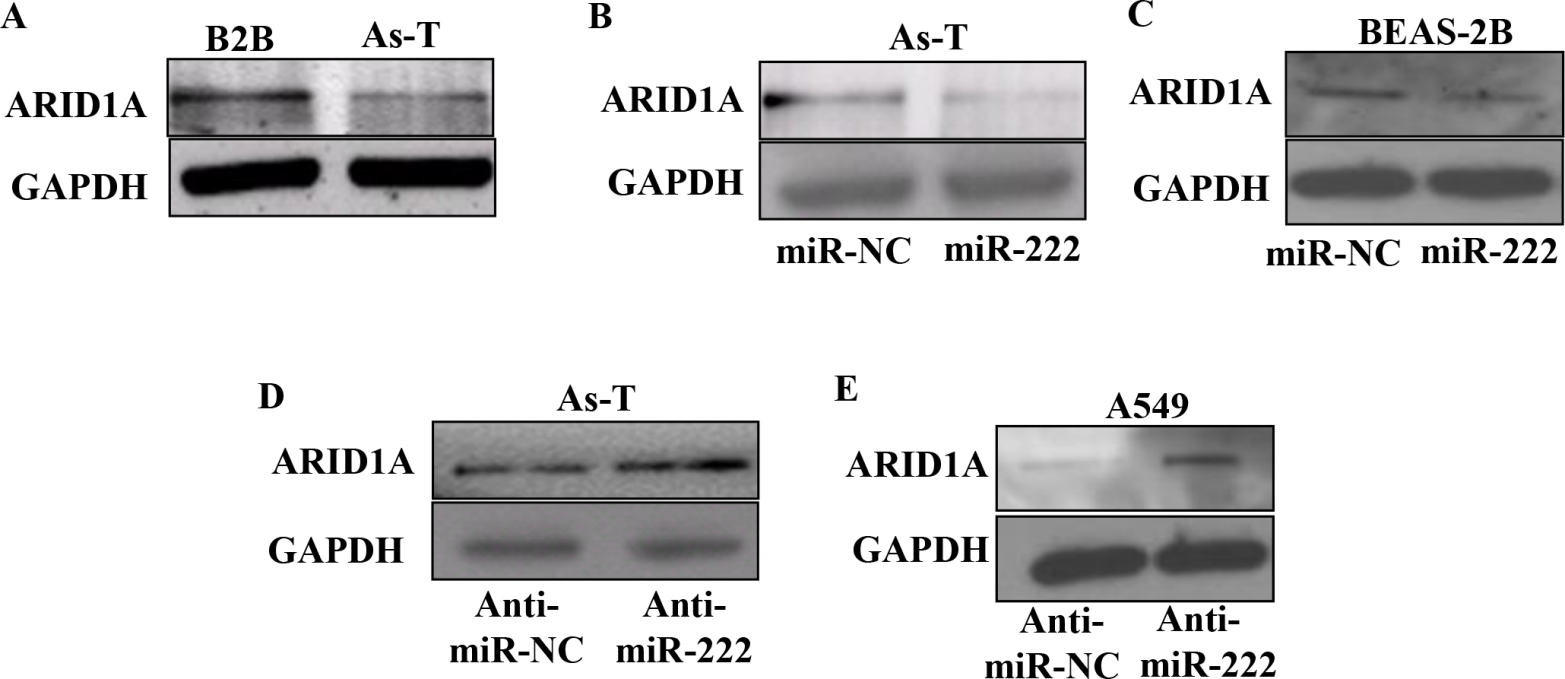
MiR-222 treatment inhibits ARID1A protein expression (**A**) Total proteins from As-T and B2B cells were used to determine protein levels of ARID1A using Western blotting. (**B**) As-T cells and (**C**) BEAS-2B cells were transfected using miR-NC or miR-222 mimic, and the expression levels of ARID1A protein in the cells were detected using Western blotting 48 h after the transfection. (**D**) As-T cells and (**E**) A549 cells were transfected using anti-miR-NC or anti-miR-222 inhibitor, and analyzed as above.

Furthermore, we used software to predict the potential targets of miR-222 and found that ARID1A was one of the putative targets of miR-222. The seed sequence of miR-222 matched 3′-UTR region of ARID1A. We constructed luciferase reporter plasmids containing the putative wild-type binding sites (WT) and seed sequence mutant sites (mut) at 3′-UTR of ARID1A (Figure 5A) and verified by sequencing. Mimics of miR-222 significantly inhibited the luciferase activity of wild-type ARID1A 3′-UTR reporter in As-T cells and BEAS-2B cells, but did not affect that of ARID1A 3′-UTR mutant reporter with the mutant binding site (Figure 5B and 5C). In the parallel experiment, inhibition of miR-222 using anti-miR-222 inhibitor increased the luciferase activity of wild-type ARID1A 3′-UTR reporter, but not that of the mutant reporter in both As-T and A549 cells (Figure 5D and 5E). We found that As-T cells have much lower levels of ARID1A protein compared to BEAS-2B cells (Figure 6A). To test the effect of miR-222 in affecting ARID1A protein expression, we showed that miR-222 mimics markedly reduced ARID1A protein levels in both As-T cells and BEAS-2B cells using Western blotting analysis (Figure 6B and 6C), while anti-miR-222 inhibitor greatly increased ARID1A protein expression in both cells (Figures 6D and 5E).

### Treatment of cells using anti-miR-222 inhibitor induced apoptosis of As-T cells through inhibiting AKT activation and inducing ARID1A expression

**Figure 7:**
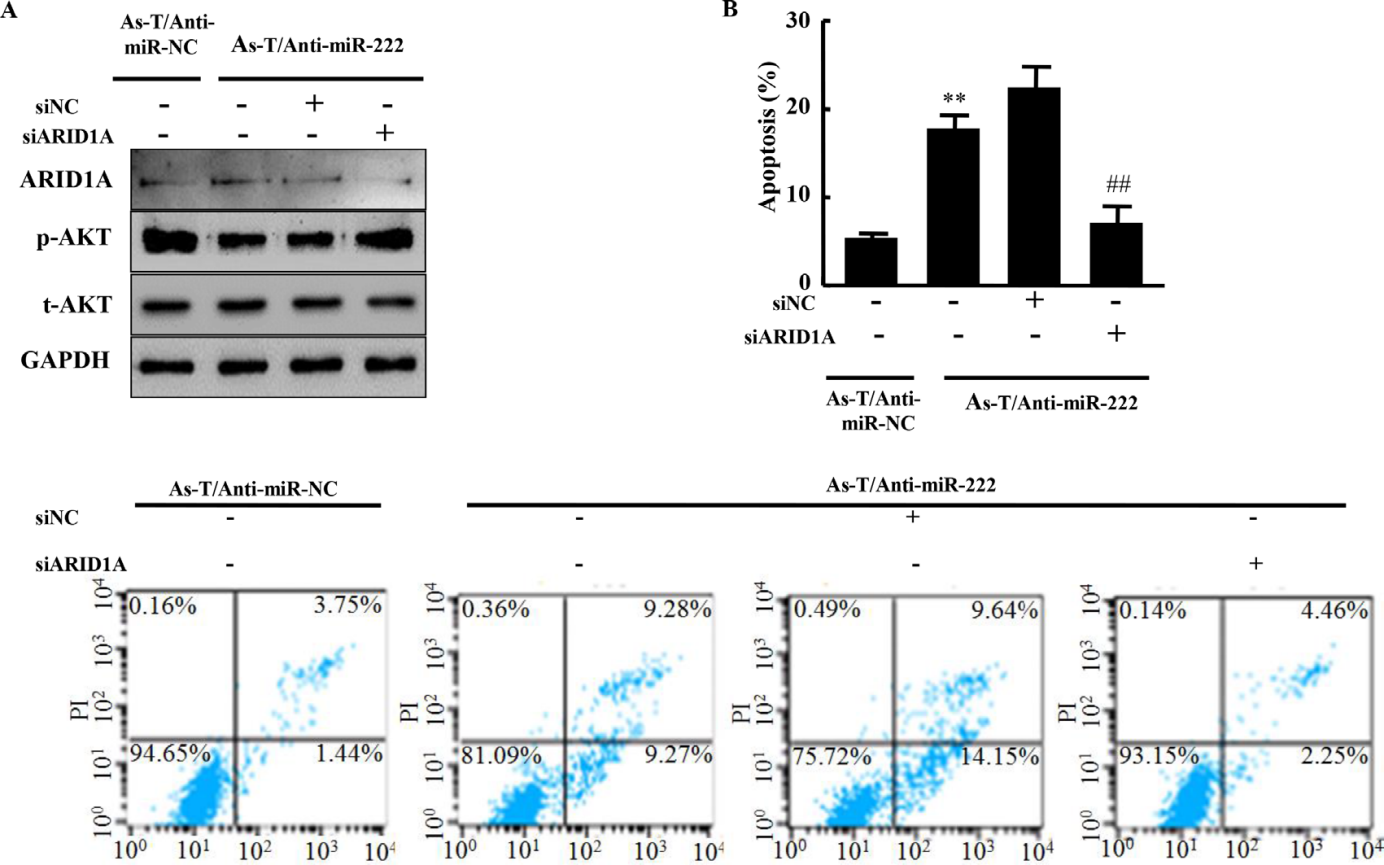
Anti-miR-222 inhibitor treatment inhibits AKT activation and induces apoptosis through ARID1A expression (**A**) As-T/anti-miR-NC, As-T/anti-miR-222 cells, and As-T/anti-miR-222 cells transfected with ARID1A siRNAs or negative control siRNA were cultured for 48 h, and analyzed using specific antibodies as indicated. (**B**) These cells were also analyzed for apoptosis using flow cytometry (FACSCanto II, BD Biosciences) and FlowJo software. The data were from three different experiments in triplicate. **indicates significant difference compared to cells without miR-222 overexpression and ARID1A knockdown. ## indicates significant difference compared to ARID1A knockdown in miR-222 overexpressing cells.

AKT is a known anti-apoptosis protein. As-T cells were transfected using anti-miR-222 inhibitor with or without ARID1A siRNAs. AKT activation (p-AKT level) is inhibited by anti-miR-222 inhibitor (Figure 7A). The ARID1A siRNAs restored p-AKT level which was reduced by anti-miR-222 inhibitor (Figure 7A). Anti-miR-222 inhibitor treatment significantly induced apoptosis, while the knockdown of ARID1A using the siRNAs reversed the effect of anti-miR-222 inhibitor in the cells (Figure 7B).

## DISCUSSION

Arsenic induces human cancers including lung cancer [19]. Recent studies have focused on arsenic-induced genetic changes, reactive oxygen species, and epigenetic alterations [20–24]. It was reported that arsenic affects the expression of certain miRNAs [15]. Increasing evidences indicate that abnormal expression of miRNAs is associated with carcinogenesis [25, 26]. In this study, we focus on the role and mechanism of miR-222 in mediating tumorigenesis using transformed cells through chronic arsenite exposure.

It was reported that the reduction of miR-222 expression inhibited cell proliferation and induced mitochondrial-mediated apoptosis of A549 lung cancer and MCF-7 breast cancer cells [27]. As an oncogene, miR-222 increased cell migration and invasion of hepatocellular carcinoma [28]. The results of the present study found that miR-222 inhibitor inhibited cell proliferation, migration, and tumor growth, indicating that miR-222 plays an important role in tumor development.

It was found that miR-222 induced gastric carcinoma cell proliferation and radioresistance by targeting PTEN [9]. The tumor suppressor PTEN is a phosphatidylinositol (PtdIns) phosphatase that regulates the activation of AKT via PtdIns 3 kinase (PI3K) to regulate cell proliferation, migration and angiogenesis [29–31]. Upregulation of PI3K/AKT/mTOR pathway is involved in the tumorigenesis of ovarian clear cell carcinoma [32], vulvar cancer [33], and other cancers particularly through mutations and inactivation of PTEN. In this study, we found that miR-222 directly targets PTEN for inducing the activation of its downstream molecules AKT and ERK in arsenic-transformed cells. In addition, we found that ARID1A is a new direct target of miR-222. ARID1A is a subunit of the SWItch/sucrose nonfermentable (SWI/SNF) chromatin remodeling complex. It has been found that low expression of the ARID1A protein occurs in high frequency in many cancers and considered as a bona fide tumor suppressor [32, 34]. Loss of ARID1A protein expression is frequently associated with PI3K/AKT pathway activation by which to promote the development of cancer, especially endometriosis-associated ovarian cancers [35]. In this study, we found anti-miR-222 inhibitor treatment in cells suppressed AKT activation and induced apoptosis through ARID1A expression in As-T cells. These results indicate that miR-222 and its novel target ARID1A play an important role in tumor growth and apoptosis, which will be helpful for understanding new mechanism of arsenic in inducing carcinogenesis and for developing new treatment option for cancer(s).

## MATERIALS AND METHODS

### Cell culture and generation of stable cell lines

Human bronchial epithelial BEAS-2B (BEAS-2B) cells were transformed into arsenic-transformed cells (As-T) by exposing BEAS-2B cells to 1 μM sodium arsenic for 26 weeks as we previously described [17]. BEAS-2B cells were cultured for 26 weeks as passage-matched cells (named as B2B). BEAS-2B and arsenic transformed (As-T) cells were cultured in Dulbecco's modified Eagle's medium (DMEM; Invitrogen, Carlsbad, CA) supplemented with 10% fetal bovine serum (FBS). Human umbilical vein endothelial cells (HUVEC, purchased from ATCC, USA) were cultured in endothelial basal medium-2 complete medium. As-T cells stably overexpressing miR-222 inhibitor or anti-miR-control were generated by infecting As-T cells with lentivirus carrying miR-222 inhibitor and green fluorescence protein (GFP) or a negative control miRNA precursor and GFP (purchased from Shanghai Genechem Co., China), followed by the selection with puromycin.

### Isolation of total RNAs, RT-PCR, and qRT-PCR analysis

Total RNAs were extracted from cultured cells using TRIzol reagent (TaKaRa, Dalian, China) according to the manufacturer's instruction. To measure the expression levels of miR-222, total RNAs were transcribed by stem-loop reverse transcription (RT) primer using PrimeScript RT Reagent Kit (TaKaRa, Dalian, China). Polymerase chain reaction (PCR) and quantitative PCR (qPCR) were performed using PCR Mix (TaKaRa, Dalian, China) and SYBR Premix DimerEraser (TaKaRa, Dalian, China) on Bio-Rad PTC-200 and ABI 7900HT system using the protocol provided by TaKaRa. U6 levels were used as an internal control. The primers are listed as followed: miR-222-StemLoop-RT primers, 5′-GTCGTATCCAGTGCAGGGTCCGAGGTATTCGCACTGGATACGACGAGACC-3′; miR-222 qPCR primers: sense, 5′-AGCTACATCTGG CTACTGG-3′, antisense: 5′-GTATCCAGTGCAGGGTCC-3′; U6-StemLoop-RT, 5′-AACGCTTCACGAATTTGCGT-3′; U6 qPCR primers: Sense, 5′-CTCGCTTCGGCAGCACA-3′, antisense, 5′-TGGTGTCGTGGAGTCG-3′.

### Cell proliferation assay

Cells at 1000 per well were seeded onto a 96-well plate and incubated at 37°C in 10% Cell Counting Kit-8 (Dojindo, Kumamoto, Japan) diluted in DMEM. Cell proliferation rates were determined at 12, 24, 48, 72, 96, and 120 h after transplantation.

### Tube formation assay

Tube formation assay (angiogenesis assay *in vitro*) was performed as we previously described [17].

### Transwell migration assay

Transwell chambers (BD Biosciences, Bedford, MA) with 8-μM pore size PET membrane were inserted into 24-well plates. About 5 × 10^4^ cells per well in DMEM without serum were seeded onto each upper well of transwell chambers, while the medium with 10% FBS as a chemoattractant was added to the lower chamber. Following incubation for 24 h at 37°C, cells that did not migrate through the membrane were removed using a cotton swab. Cells that had migrated through the membrane were fixed with 20% methanol and stained with 0.1% crystal violet (Sigma, Saint Louis, MO, USA), imaged, and counted.

### Tumor formation experiment

Ten 5- to 6-week-old BALB/c nude mice per group were subcutaneously injected with 5 × 10^6^ As-T cells infected with lentiviruses carrying miR-NC or miR-222 inhibitor in special pathogen-free conditions. Tumor sizes were measured twice every week using Vernier caliper after they were visible. Tumor volumes were calculated according to the formula 0.5 × length × width^2^ [18].

### Luciferase assay

3′-Untranslated region (3′-UTR) wild-type or mutant-type reporter of PTEN was constructed as previously reported [9]. The 3′-UTR region of AT-rich interactive domain 1A (ARID1A) was amplified using PCR from cDNAs derived from As-T cells, and inserted into pMIR-REPORTER vector (Thermo Scientific, Rockford, IL, USA). Primers for wild-type or mutant reporter constructs are listed as followed: ARID1A-WT-sense, 5′GTTTTAGCTACATTGTTGAAATACCCAAAGCTTGGG-3′, Antisense, 5′-GGACTAGTCCTGAATAAATGATATTCATTAAGCC-3′; ARID1A-MT-sense, 5′GTTTTATTGAAATTGTTGAAATACCCAAAGCTTGGG-3′, antisense: 5′-GGACTAGTCCTGAATAAATGATATTCATTAAGCC-3′. As-T cells at 1 × 10^5^ per well were plated onto 24-well plates for 12 h before the transfection. As-T cells were transiently cotransfected with 300 ng of wild type or mutant PTEN-3′-UTR reporter, or 300 ng of wild type or mutant ARID1A-3′-UTR reporter, 100 ng pGl4.74, and 20 nM miR-222 mimic or anti-miR-222 inhibitor using 1.5 μL Lipofectamine reagent (Invitrogen). Then firefly and Renilla luciferase activities were measured by the Dual-Luciferase Reporter Assay System (Promega, WI, USA) on BIOTEK synegy2 Luminometer. Renilla luciferase activities were normalized to firefly luciferase activities for transfection efficiency 48 h after the transfection.

### Western blotting analysis

The cells were washed twice with ice-cold PBS buffer, and total protein lysates were prepared using radioimmunoprecipitation assay buffer supplemented with protease inhibitors (Beyotime, Nantong, China). Aliquots of protein lysates (30 μg) were electrophoresed on 8% (for ARID1A) or 10% sodium dodecyl sulfate-polyacrylamide gels (for other proteins), and transferred to a nitrocellulose membrane (Roche, Switzerland). Next, the membranes were incubated with primary antibodies purchased from Cell Signaling Technology (Danvers, MA, USA) overnight at 4°C, then with the appropriate horseradish peroxidase-conjugated secondary antibody. Immunoreactivity signals were visualized using chemiluminescence detection reagent and imaged.

### Apoptosis assay by flow cytometry

Apoptosis of cells was assessed using Annexin V-FITC apoptosis kit according to the manufacturer's instructions (BD Pharmingen, USA). The cells were then analyzed using flow cytometer (FACSCanto II, BD Biosciences) after the cells were stained with anti-Annexin V-FITC antibody. The data were analyzed using FlowJo software. Three experiments were performed in triplicate.

### Statistical analysis

Statistical analysis was performed using Microsoft Excel 2007. Data are presented as mean ± standard deviation or as mean ± standard error of mean, as indicated, and two-tailed Student's *t*-test was used for comparison with *indicates significant difference at *P* < 0.05 and **indicates significant difference at *P* < 0.01.

## SUPPLEMENTARY MATERIALS FIGURE



## Abbreviations

miRNAs: microRNAs
B2B: BEAS-2B
As-T: arsenic-transformed human lung epithelial BEAS-2B
CCK-8: Cell Counting Kit-8
PTEN: phosphatase and tensin homolog deleted on chromosome 10
ARID1A: AT-rich interactive domain 1A
RT-PCR: reverse transcription-polymerase chain reaction
RT-qPCR: reverse transcription-quantitative polymerase chain reaction
SE: standard error
snRNA: small nuclear RNA
