# Supplementary material for: Role and mechanism of miR-222 in arsenic-transformed cells for inducing tumor growth
Source: Oncotarget. 2016 Feb 20;7(14):17805–14. doi: 10.18632/oncotarget.7525 (PMC4951251; doi:10.18632/oncotarget.7525)
Supplement: Supplementary file 1 [file oncotarget-07-17805-s001.pdf]

Role and mechanism of miR-222 in arsenic-transformed cells for inducing tumor growth

Supplementary Materials

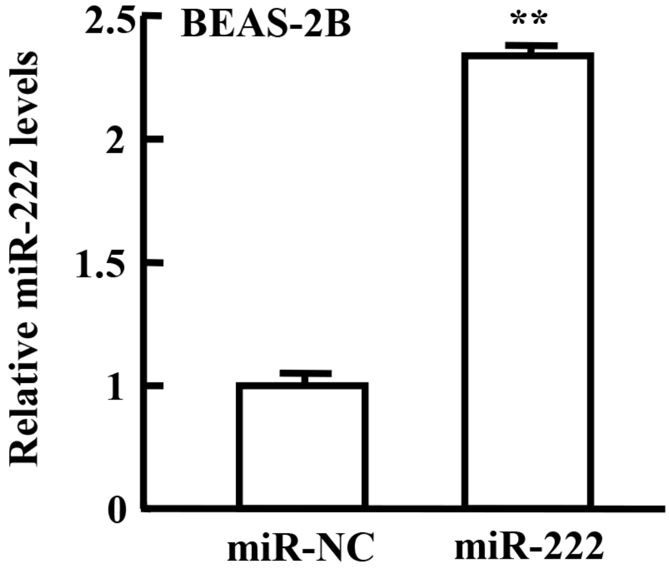

Supplementary Figure S1: miR-222 levels were increased in BEAS-2B cells transfected with miR-222 mimics. BEAS-2B cells were transfected with miR-NC or miR-222 mimic. The levels of miR-222 in these cells were analyzed using RT-qPCR 48 h after the transfection. **indicates significant difference compared to the control cells ($P < 0.01$).
